# Supplementary figures and images for: A novel mouse model of obstructive sleep apnea by bulking agent-induced tongue enlargement results in left ventricular contractile dysfunction
Source: PLoS One. 2020 Dec 10;15(12):e0243844. doi: 10.1371/journal.pone.0243844 (PMC7728202; doi:10.1371/journal.pone.0243844)

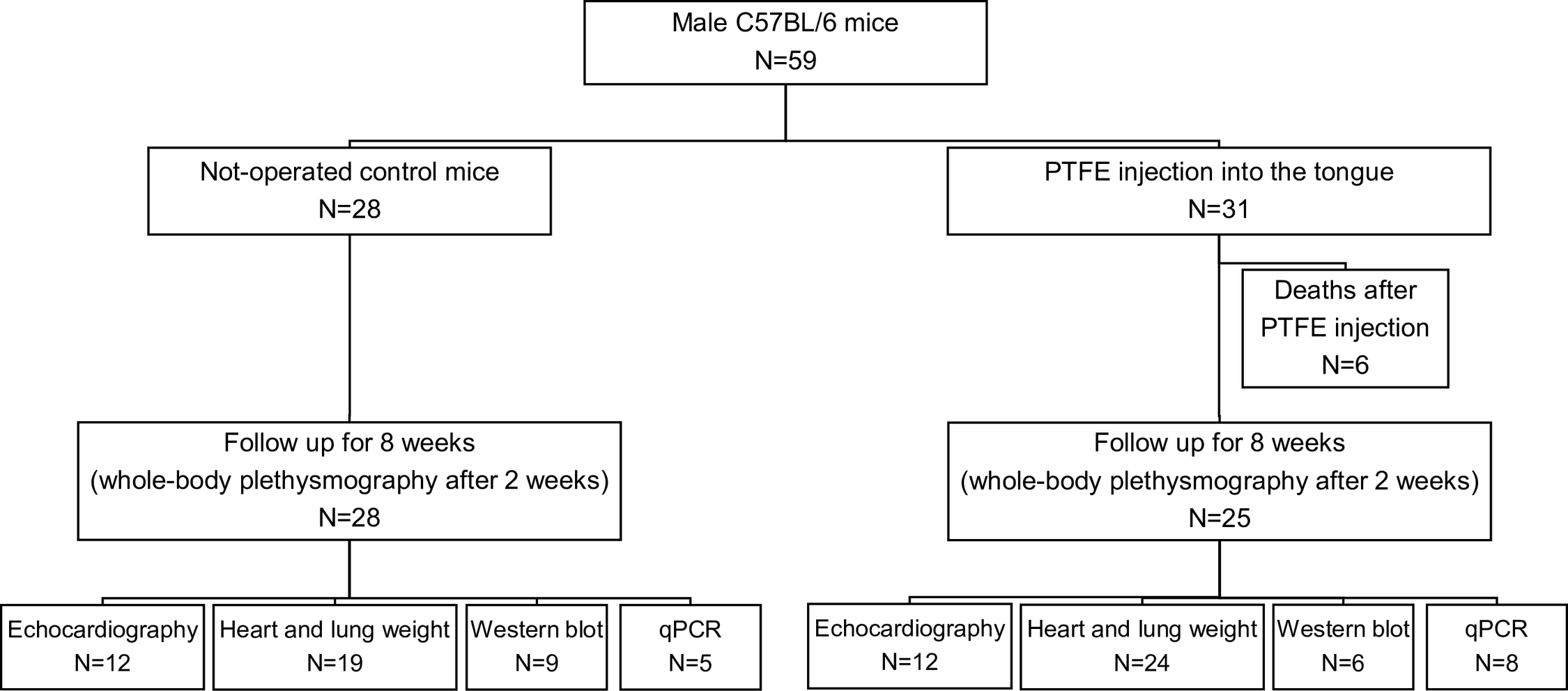

Supplement: S1 Fig — Study flowchart showing the allocation of 59 mice in total. 31 mice were subjected to tongue enlargement by PTFE and 28 littermates were used as control animals. 16 mice (5 control vs. 11 PTFE mice) were used for some proof of principle experiments that are part of the Supporting information. Since they are not part of the main manuscript, they are not shown in this study flowchart. (TIF) [file pone.0243844.s001.tif]

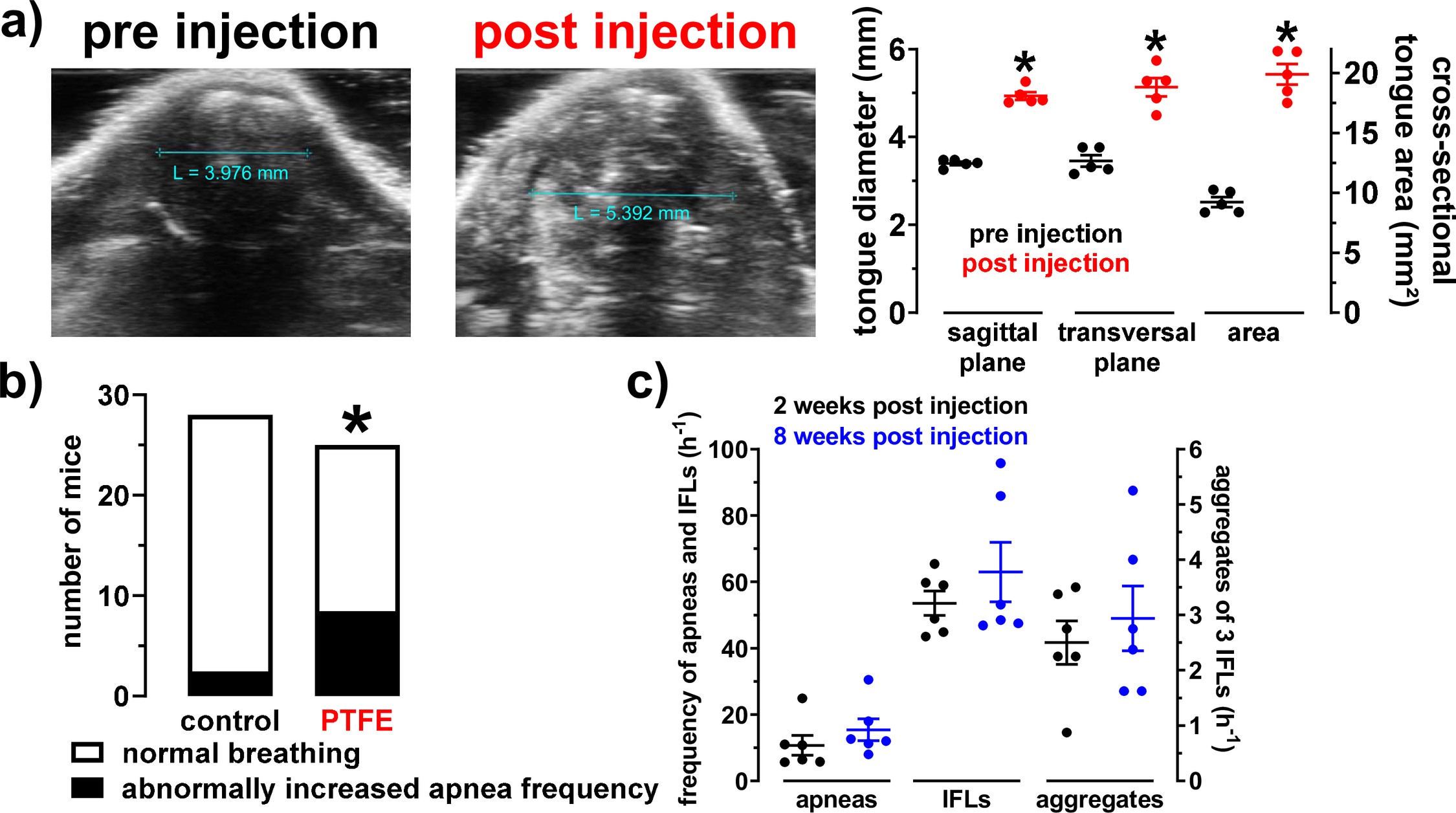

Supplement: S2 Fig — a) Original ultrasound image of a murine tongue in transversal plane before and after (PTFE) injection (left panel). Interestingly, we observed a strong increase in the lateral tongue diameter. The mean data for 5 animals is shown in right panel. PTFE injection resulted in a significant increase in both lateral (transversal plane) and dorso-ventral (sagittal plane) tongue diameters. Cross-sectional tongue area was calculated by lateral diameter*dorso-ventral diameter*0.25*π estimating an elliptical shape of the tongue. b) After PTFE injection, the proportion of mice showing an abnormally increased apnea frequency (cut-off 14.75 apneas/h) was significantly increased. c) Importantly, frequencies of apneas, IFLs, and IFL aggregates remained stable for the whole 8-week observation period (N = 6). *—P<0.05 vs. pre injection (a) or control (b), one-way repeated measures ANOVA with Holm-Sidak’s post-hoc correction (a+c) and Chi-square test (b). (TIF) [file pone.0243844.s002.tif]

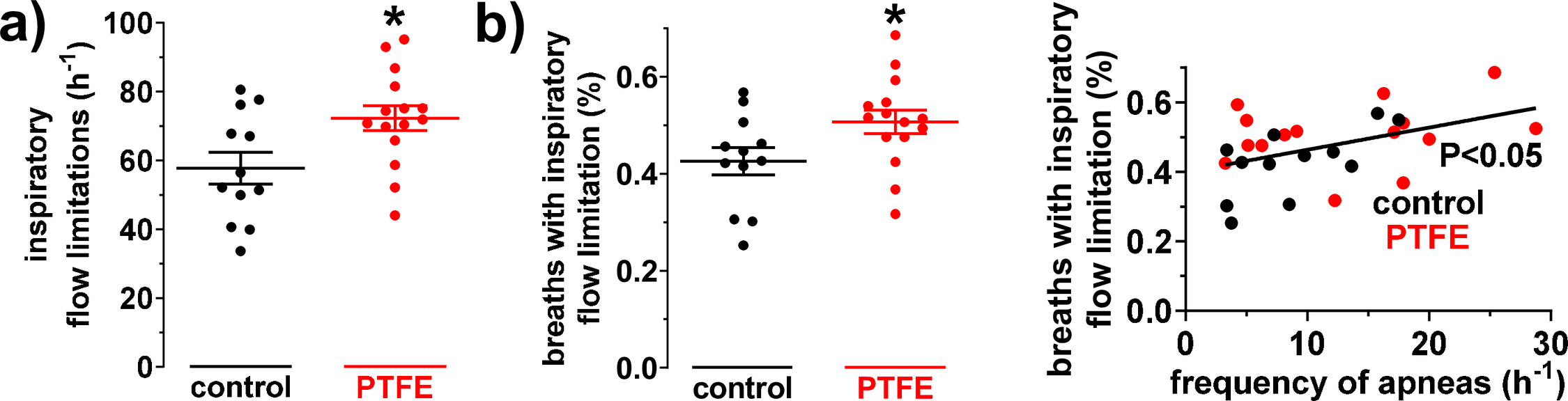

Supplement: S3 Fig — a) PTFE-injected mice showed a significant increase in the absolute frequency of inspiratory flow limitations (IFLs/h). b) In accordance, we observed a significantly increased percentage of flow limited breaths in PTFE mice that also correlated significantly positive with the frequency of apneas. *—P<0.05, Student’s t-test and linear regression analysis, as appropriate. (TIF) [file pone.0243844.s003.tif]

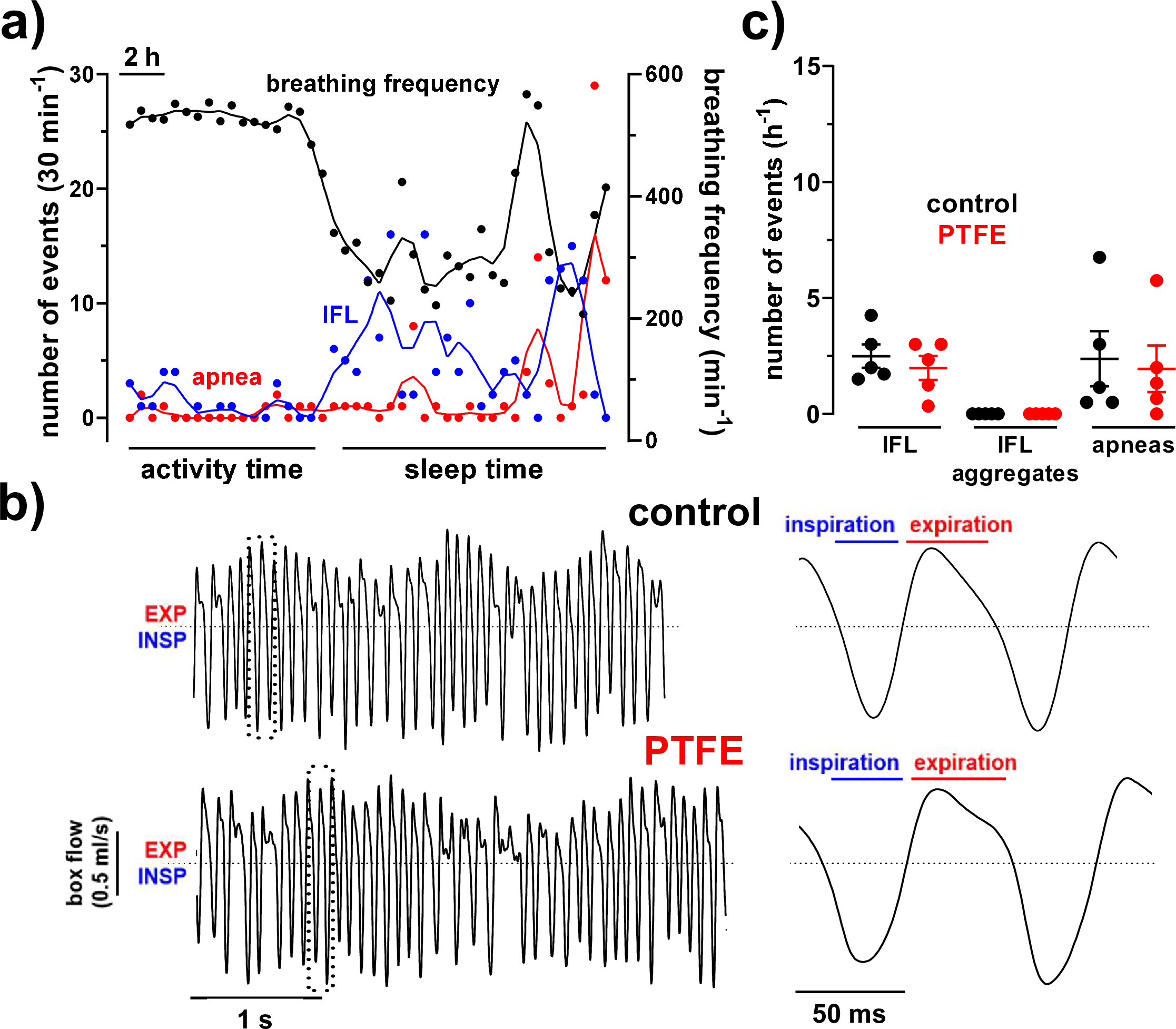

Supplement: S4 Fig — a) Average breath frequency, and the number of IFL and apneas were calculated for 30 min intervals during a 22 h observation period (from 10 p.m. to 8 p.m. the other day) in a PTFE-treated mouse. Time of activity of conscious mice can be easily discriminated from sleep time by monitoring average breathing frequency. An increased number of IFLs was typically accompanied by a concomitant increase in the number of apneas only during sleep time. b) Original box flow recordings of a control (upper panel) and a PTFE-treated mouse (lower panel) measured by whole-body plethysmography in conscious mice. Both mice showed a similar breathing pattern, indicating that tongue enlargement due to PTFE injection does not induce upper airway obstruction in conscious mice. c) Mean data for IFL and apnea frequency during activity time. In conscious mice (activity time at night), a negligible number of apneas and IFL could be detected with no difference between PTFE-treated and control animals. Kruskal-Wallis test with Dunn’s post-hoc correction. (TIF) [file pone.0243844.s004.tif]

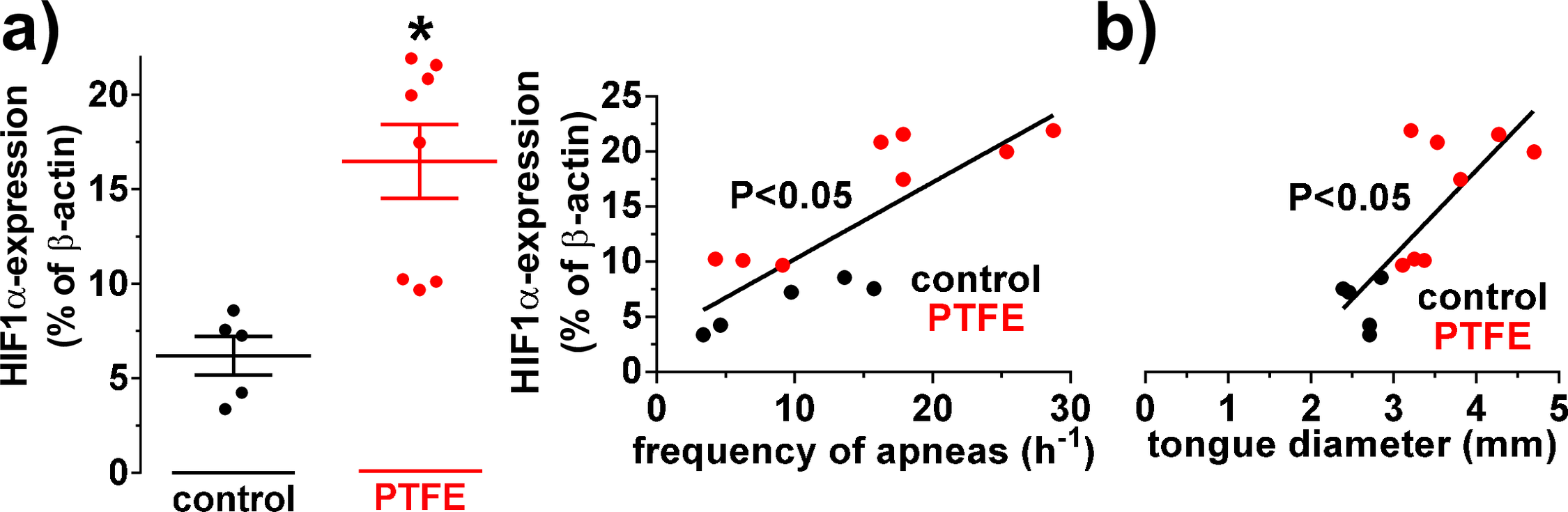

Supplement: S5 Fig — HIF1α mRNA expression was analyzed by qPCR (normalized to β-actin) from hearts. a) Scatter plots of HIF1α mRNA expression in control (N = 5) and PTFE-treated (N = 8) animals (left panel). There was a significant upregulation of HIF1α mRNA expression after PTFE treatment. The level of HIF1α mRNA expression correlated significantly with the frequency of apneas (right panel). b) Interestingly, the tongue diameter correlated significantly positive with the HIF1α expression, indicating hypoxemia due to PTFE-dependent tongue enlargement. *—P<0.05, Mann-Whitney test and linear regression analysis, as appropriate. (TIF) [file pone.0243844.s005.tif]

CaMKII

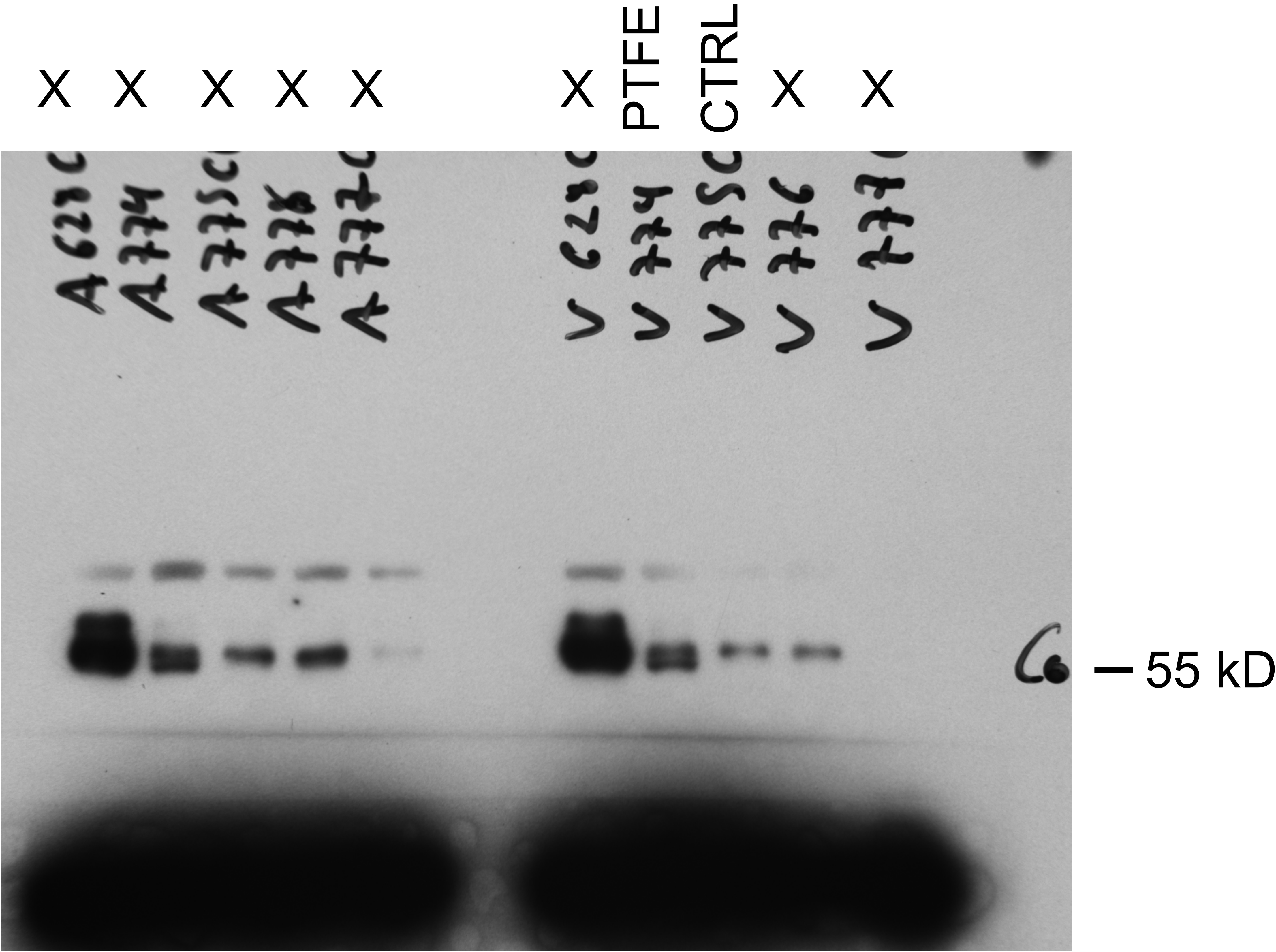

GAPDH

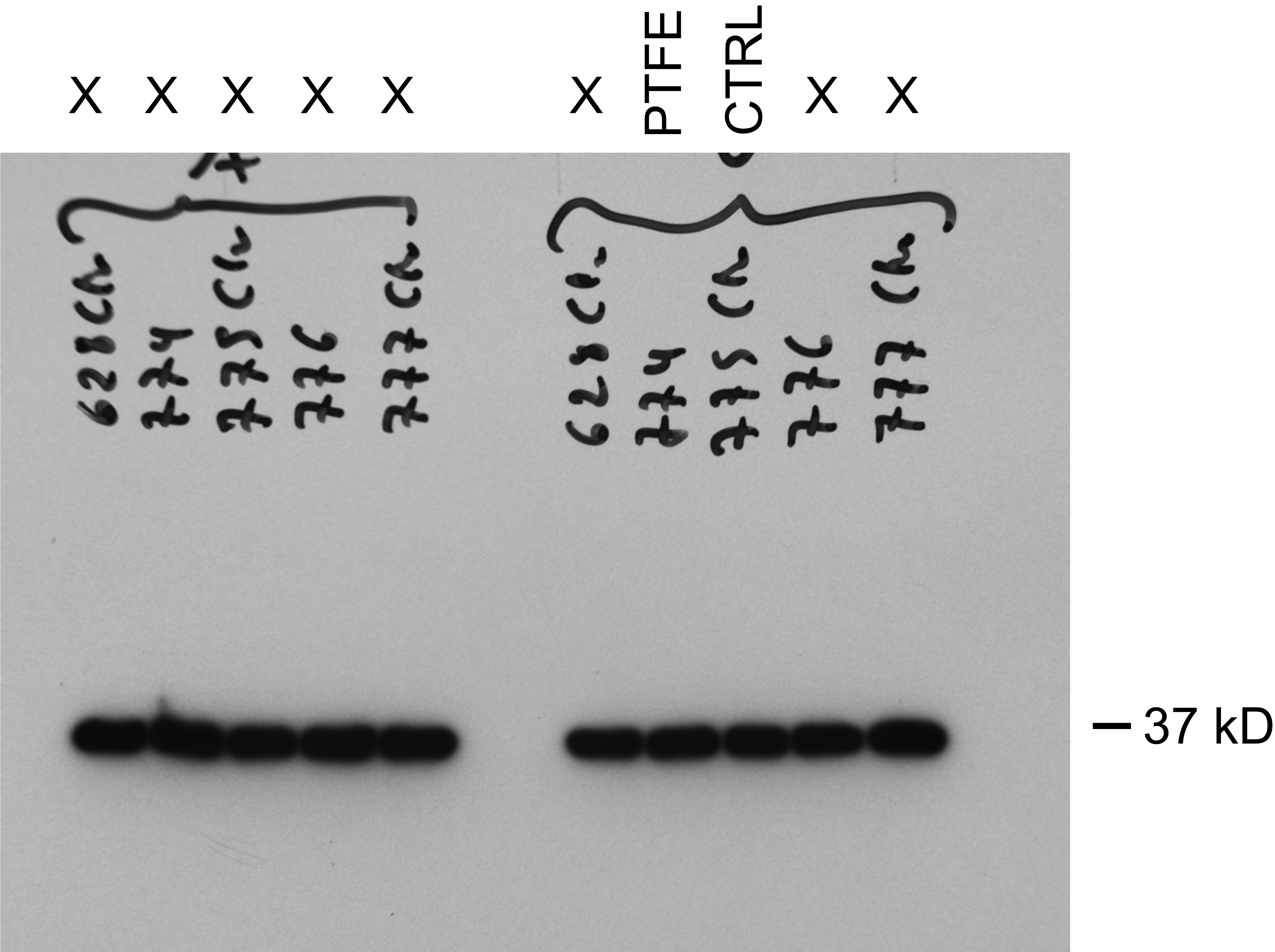

Supplement: S1 Raw images — (PDF) [file pone.0243844.s006.pdf]
